# The Plant Growth-Promoting Fungus (PGPF) *Alternaria* sp. A13 Markedly Enhances *Salvia miltiorrhiza* Root Growth and Active Ingredient Accumulation under Greenhouse and Field Conditions

**DOI:** 10.3390/ijms19010270

**Published:** 2018-01-16

**Authors:** Li Si Zhou, Kun Tang, Shun Xing Guo

**Affiliations:** Institute of Medicinal Plant Development, Chinese Academy of Medical Sciences & Peking Union Medical College, Beijing 100193, China; zls921203@sina.com (L.S.Z.); cqupttk@163.com (K.T.)

**Keywords:** bio-fertiliser, *Alternaria*, *Salvia miltiorrhiza*, lithospermic acid B (LAB), cinnamic acid 4-hydroxylase (C4H)

## Abstract

Plant growth-promoting fungi (PGPF) have attracted considerable interest as bio-fertilisers due to their multiple beneficial effects on plant quantity and quality and their positive relationship with the ecological environment. Advancements in the development of PGPF for crops and economic plant cultivation applications have been achieved, but such improvements for the use of PGPF with popular medicinal herbs, such as *Salvia miltiorrhiza*, are rare. In this study, we collected *S. miltiorrhiza* specimens inhabiting wild, semi-wild, farmland and pot-cultured areas in the Henan province of China and isolated endophytes from the roots, shoots and leaves of these samples. Twenty-eight strains of the dominant genus *Alternaria* were identified and selected as candidate PGPF. Under greenhouse conditions, *Alternaria* sp. A13 simultaneously enhanced the dry root biomass and secondary metabolite accumulation of *S. miltiorrhiza* as the optimal PGPF of the 28 candidate isolates. To further assess the interaction between *S. miltiorrhiza* and *Alternaria* sp. A13, the effects on seedlings growth, active ingredient accumulation, and the activity of key enzymes for effective biosynthetic pathways were investigated over a period of six months under field conditions. Compared to uninoculated seedlings, *S. miltiorrhiza* seedlings colonised by *Alternaria* sp. A13 showed significant increment of 140% in fresh weight, 138% in dry weight, and enhancement in the contents of total phenolic acid, lithospermic acids A and B (LAA and LAB, respectively) of 210%, 128% and 213%, respectively. Examination of the related enzyme activities showed that the elicitation effect of A13 on LAB accumulation correlated with cinnamic acid 4-hydroxylase (C4H) activity in the phenylpropanoid pathway under field conditions. Our results confirmed that *Alternaria* sp. A13 not only contributes to the stimulation of *S. miltiorrhiza* root growth, but also boosts the secondary metabolism, thus demonstrating its application potential as a bio-fertiliser for *S. miltiorrhiza* cultivation, especially in areas outside of its native growth regions.

## 1. Introduction

*Salvia miltiorrhiza* is a herbaceous perennial plant of the family Lamiaceae native to central and north-eastern China. The roots of *S. miltiorrhiza* have been used in traditional Chinese medicine for thousands of years. The plant is documented as an extremely effective herb for the treatment of cardiodynia in the first official book on traditional Chinese herbal drugs [1] and is still widely used in the clinical treatment of blood circulation diseases. With increasing stress, based on fast-paced lifestyles and global environmental deterioration, cardiovascular diseases cause 17.5 million deaths each year and are the main cause of death worldwide [2] (November 2016). Accordingly, the increased demand for *S. miltiorrhiza* and its active compounds exceeds the existing industrial and agricultural supplies. *Salvia miltiorrhiza* has been successfully introduced and cultivated in non-origin countries, such as Germany [3], but the effects of these different cultivation conditions on the quality of *S. miltiorrhiza* have, so far, not been characterised. According to the traditional Chinese medicinal prescription, decoction is the main method for consuming *S. miltiorrhiza* root compounds. Therefore, the active ingredients responsible for the curative effects of *S. miltiorrhiza* roots should be water-soluble phenolic acids, which have received considerable attention in recent years [4,5]. There are two parallel pathways for initial phenolic acids biosynthesis: the tyrosine-derived pathway and the phenylpropanoid pathway [6]. Phenylalanine ammonia-lyase (PAL) and cinnamic acid 4-hydroxylase (C4H) are believed to catalyse the first two steps, which begin with l-phenylalanine. Tyrosine aminotransferase (TAT) and 4-hydroxyphenylpyruvate reductase (HPPR) catalyse the first two enzymatic steps in the tyrosine-derived pathway.

Due to the significant interest in the metabolites of *S. miltiorrhiza* roots as raw materials for pharmaceuticals, studies have increasingly focused on methods to enhance the production of these active metabolites, including the development of novel strains that can produce the active compounds [7] as well as the use of biotic and abiotic elicitors to stimulate the accumulation of active metabolites in cell or hairy root cultures of *S. miltiorrhiza* [4,8,9,10]. However, these solutions have not been fully developed and applied in large-scale production, and extraction from field-cultivated *S. miltiorrhiza* roots remains the major source of bioactive compounds. Therefore, *S. miltiorrhiza* cultivation urgently requires new methods to improve field management measures to enhance the growth and secondary metabolite accumulation of this medicinal plant. Plants benefit substantially from plant–microbe symbiotic systems, and some plant growth-promoting endophytes have been applied as potential bio-fertilisers in the cultivation of economic crops [11], such as rice seedlings inoculated with *Pseudomonas stutzeri* A15 [12] and maize, potato, cucumber and tomato plants inoculated with *Paenibacillus polymyxa* CR1 [13]. Plant growth-promoting fungi (PGPF) that promote higher yields and induce secondary metabolite accumulation are optimal for medicinal herb plants. Live *Piriformospora indica* has been used to enhance asiaticoside production in tissue culture seedlings of *Centella asiatica* [14], while live *Mycena* sp. F-23 has been used to stimulate the kinsenoside and flavonoid contents of *Anoectochilus formosanus* in pot cultures [15]. Thus, the application of PGPF as bio-fertilisers in medicinal herb cultivation is promising and has benefits that include improving the quantity and quality of products as well as reducing the pollution in the agricultural environment due to chemical fertilisers.

Wang [16] found that the effective components of *S. miltiorrhiza* roots are related to the diversity and quantity of the habituated endophytic fungi of the plant. Consequently, endophytic or rhizosphere microorganisms derived from *S. miltiorrhiza* grown in native producing areas might be excellent candidate plant growth-promoting microbes for application in *S. miltiorrhiza* cultivation. However, most previous studies have focused on promoting the growth of hairy root cultures [8,17]. Research on the application of PGPF in *S. miltiorrhiza* cultivation is extremely limited. Analysis of the diversity of endophytic fungi derived from *S. miltiorrhiza* grown in native producing areas [18] has shown that *Alternaria* was the dominant fungus, consistent with the findings of Yan [19]. In addition, many strains in the genus *Alternaria* produce the same secondary metabolites as their host plants, e.g., paclitaxel in *Alternaria alternate* TPF6 [20], podophyllotoxin and its analogues in *Alternaria neesex* Ty [21], vinblastine in *Alternaria* sp. 97CG1 [22] and flavonoids in *Alternaria tenuissima* Y2-3 [23]. Thus, screening endophytic fungi of the genus *Alternaria* isolated from *S. miltiorrhiza* grown in native producing areas would be a reliable and effective way to identify PGPF that promote host medicinal plant growth and secondary metabolite accumulation.

In this study, we identified and selected 28 *Alternaria* candidates isolated from *S. miltiorrhiza* that inhabited native wild, semi-wild and farmland areas to screen for PGPF to apply to *S. miltiorrhiza* plants. We then evaluated the effects of the isolated PGPF on *S. miltiorrhiza* growth and secondary metabolite accumulation under greenhouse conditions. Finally, the most effective endophytic fungi were further tested under field conditions to examine their effects on the growth, secondary metabolite accumulation and phenolic acid biosynthetic enzyme activities of *S. miltiorrhiza* over a period of six months.

## 2. Results

### 2.1. Screening for the Optimal PGPF under Greenhouse Conditions

**Figure 1:**
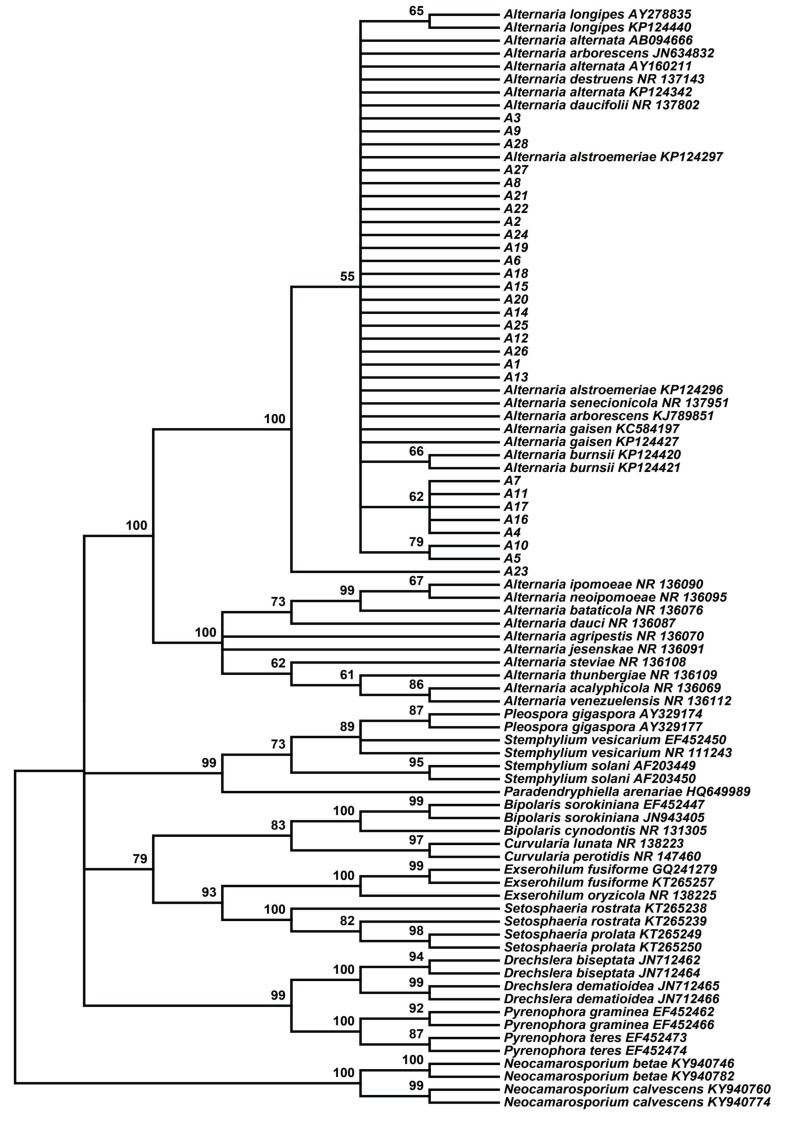
Tree topology of 28 strains based on their internally transcribed spacer (ITS) region sequences using the Neighbour-Joining (N-J) method. The 57 reference sequences were obtained from GenBank. Bootstrap values (calculated from 1000 repetitions) ≥50% are shown at their relevant nodes.

Using fresh tissue separation methods, 64 strains with different growth phenotypes were isolated from *S. miltiorrhiza* inhabiting wild, semi-wild and farmland areas in the Henan province of China. Phylogenetic analysis of the internally transcribed spacer (ITS) regions of the ribosomal DNA (rDNA) indicated that 28 of the isolated endophytic fungi belonged to the genus *Alternaria* (Figure 1), which is the dominant genus associated with *S. miltiorrhiza*. Subsequently, to select the optimal PGPF under greenhouse conditions, the 28 candidates were evaluated for their effects on *S. miltiorrhiza* growth and active ingredient accumulation. 

**Figure 2:**
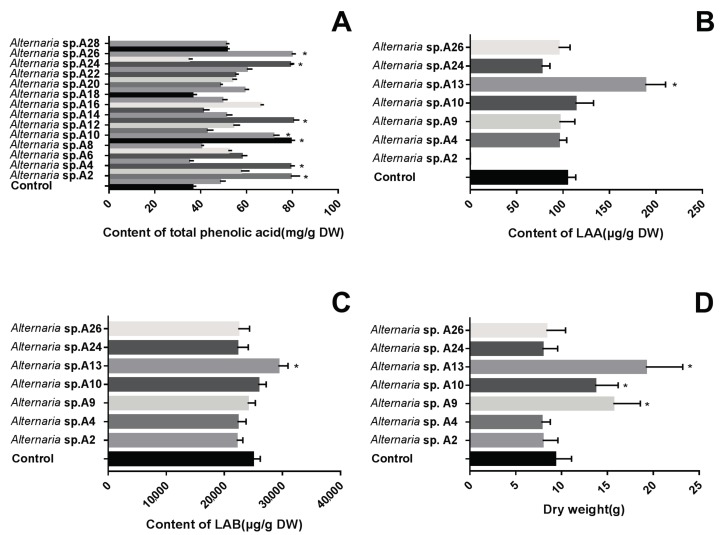
Effects of the 28 candidates on the total phenolic acid contents of *S. miltiorrhiza* (**A**) and the effects of the seven selected strains on the LAA (**B**) and LAB (**C**) content and root dry weight (**D**) of *S. miltiorrhiza* under greenhouse conditions. Vertical bars represent the SD values (*n* = 3). *Asterisks* indicate statistically significant differences between the treatment and the control (* *p* < 0.05) by Student’s *t* test.

Samples A2, A4, A9, A10, A13, A24, and A26 were notable among the 28 isolates for being effective in promoting the accumulation of total phenolic acid under greenhouse conditions (Figure 2A). The seven effective endophytic fungi were further tested with respect to their effects on the growth and bioactive metabolite accumulation of *S. miltiorrhiza* roots. Isolates A9 and A10 were less effective than A13 in terms of root dry weight, but they were more effective than the control or the other four *Alternaria* sp. (Figure 2D) isolates at simultaneously enhancing the dry biomass of the roots (Figure 2D) and increasing the contents of both lithospermic acid A (LAA; Figure 2B) and B (LAB; Figure 2C). Thus, isolate A13 emerged as the optimal PGPF among the seven effective *Alternaria* strains when applied to *S. miltiorrhiza* cultivation.

### 2.2. Verifying the Promoting Effects Induced by the Optimal PGPF in the Field Experiments

**Figure 3:**
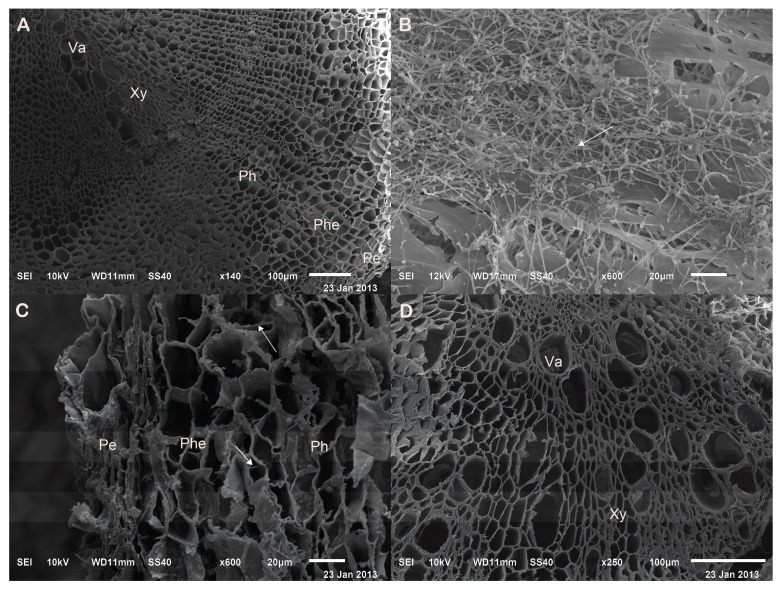
Scanning electron microscopy (SEM) images of *Salviae miltiorrhiza* roots after inoculation with A13 under field conditions. Roots of the control group (**A**); Distribution of hyphae outside the periderm (**B**); Distribution of hyphae inside the phellogen cells (**C**); Hyphae did not appear in the phloem, the xylem or the vascular cambium after A13 inoculation (**D**). Arrows indicate the hyphae of A13. Pe: periderm; Phe: phellogen; Ph: phloem; Xy: xylem; Va: vascular cambium.

According to scanning electron microscopy (SEM) observations, A13 successfully colonised the roots of *S. miltiorrhiza* (Figure 3). Compared with the control group (Figure 3A), in the experimental group, hyphae appeared inside the phellogen cells (Figure 3C) and outside the periderm (Figure 3B). Neither the control group (Figure 3A) nor the experimental group (Figure 3D) were colonized by A13 in the phloem, the xylem or the vascular cambium. The fungus colonised the periderm, with hyphae invading parallel to the root axis, producing coiled hyphae intracellularly. In addition, the phellogen cells and the phloem parenchyma cells of the colonised roots also showed abnormal alterations, such as irregular thickening of the cell walls and the accumulation of cell inclusions (Figure 3C).

**Figure 4:**
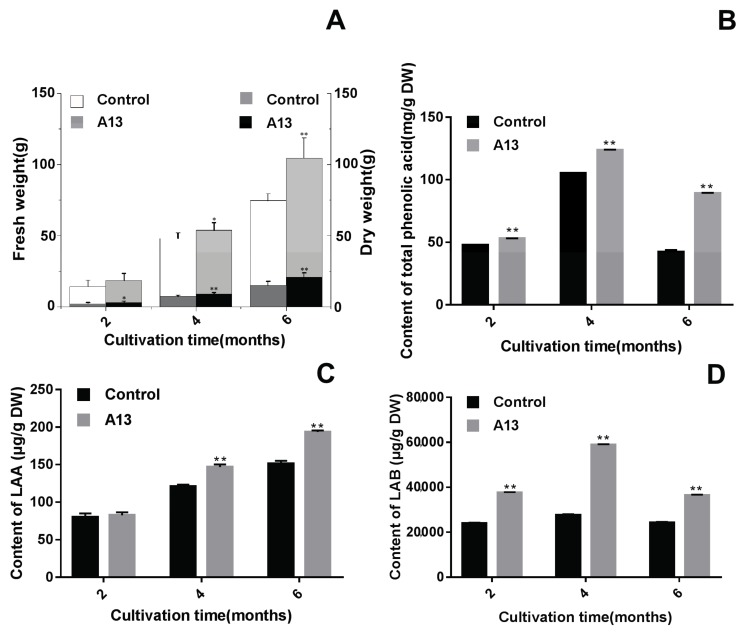
Effects of A13 on the root biomass (**A**) and accumulation of total phenolic acid (**B**), lithospermic acid A (**C**) and lithospermic acid B (**D**) content in the roots of *Salvia miltiorrhiza* under field conditions. Vertical bars represent the SD values (*n* = 10). Asterisks indicate statistically significant differences between A13 and the control (* *p* < 0.05; ** *p* < 0.01) by Student’s *t* test.

Colonisation by A13 resulted in a rapid increase in the root biomass of *S. miltiorrhiza* (Figure 4A). The enhancing effect of A13 on the fresh weight could be observed within four months. When A13 was inoculated into the host plant, root growth was promoted, increasing the fresh weight from 74.59 g in the control culture to 104.49 g in the symbiotic culture after six months of growth. Similar to the fresh weight, A13-colonised roots showed a more rapid increase in dry weight than that of the control. Inoculation of A13 into the roots at any time (two, four or six months) caused a rapid and significant increase in the dry weight from 2.94 to 20.97 g, suggesting that inoculation with A13 was beneficial for root biomass accumulation.

The time course of the effect of A13 on the accumulation of total phenolic acid, LAA and LAB in the roots of *S. miltiorrhiza* under field conditions is shown in Figure 4B–D. Rapid increases in the accumulation of total phenolic acid and LAB were observed four months after inoculation with A13, at which time both reached their peak values over the entire six-month co-culture period. In addition, over the entire cultivation period, both the total phenolic acid content and LAB accumulation in the roots treated with A13 were higher (*p* < 0.01) than those of the control. Meanwhile, the LAA content was enhanced significantly by A13 in month 4 and reached its maximum in month 6. Compared with the other three estimated parameters (root fresh weight; root dry weight; LAA) affected by A13, the changes in the total phenolic acid and LAB contents were greatest. Upon A13 induction, the total phenolic acid accumulation was 2.06-fold higher than that of the control in the previous cultivation period. The roots treated with A13 attained LAB contents of 58,898 µg/g dry weight in month 4 and 36,423 µg/g dry weight in month 6 after inoculation, which were 2.1- and 1.5-fold higher than the values of the control at the same sampling time points.

### 2.3. Influence of A13 on Phenolic Acid Biosynthetic Enzyme Activity

**Figure 5:**
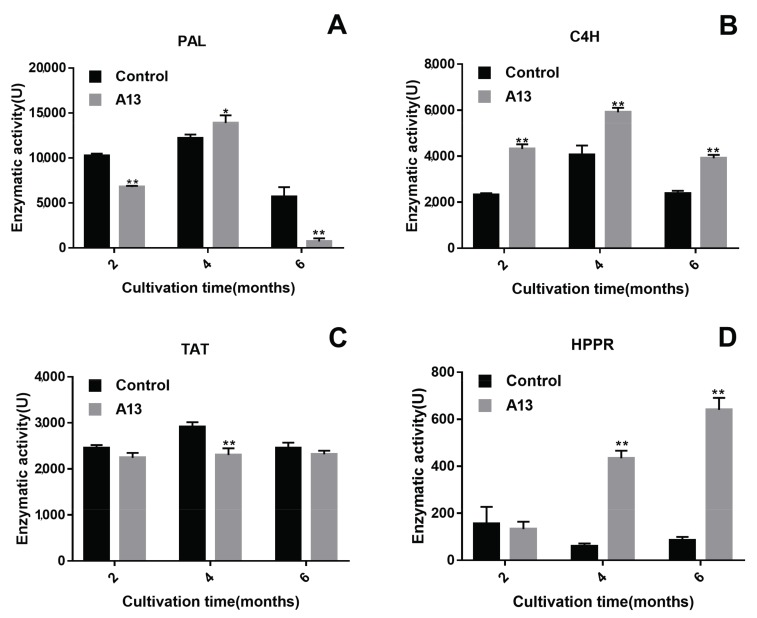
Effects of A13 on PAL (**A**), C4H (**B**), TAT (**C**) and HPPR (**D**) activity in the roots of *Salvia miltiorrhiza* under field conditions. Vertical bars represent the SD values (*n* = 3). Asterisks indicate statistically significant differences between A13 and the control (* *p* < 0.05; ** *p* < 0.01) by Student’s *t* test.

As the key enzymes of the water-soluble phenolic acid biosynthetic pathway, PAL, C4H, TAT and HPPR affect the accumulation of total phenolic acid and LAB in the roots of *S. miltiorrhiza*. Therefore, we measured the activities of these key enzymes involved in phenolic acid biosynthesis (Figure 5). Phenylalanine ammonia-lyase is the first key enzyme in the phenylalanine pathway branch of the RA biosynthetic pathway in the roots of *S. miltiorrhiza*. At the four-month sampling point, the PAL activity in the roots treated with A13 differed significantly from that in the uninoculated roots; however, this activity decreased dramatically at the six-month sampling point and was significantly lower than those of the control during the same period. The C4H catalyses the second reaction in the phenylalanine pathway branch of the RA biosynthetic pathway. At all three sampling points, C4H activity in the colonised roots was higher than that of the control. While this activity sharply decreased at the end of the culture period in both the inoculated and uninoculated roots, the C4H activity in the colonised roots was still significantly higher (1.7-fold) than that of the control. Tyrosine aminotransferase is the first enzyme in the tyrosine-derived branch of the phenolic acid biosynthetic pathway. Changes in TAT activity between the inoculated and uninoculated roots were not obvious at the two- and six-month sampling points, but they were greatly decreased at the 4-month sampling point. The HPPR catalyses the second specific biosynthetic step in phenolic acid biosynthesis from tyrosine. Colonisation of the roots by A13 had an extreme effect on HPPR activity at the 4- and 6-month sampling points and was 7.1- and 7.5-fold higher, respectively, than that of the control.

## 3. Discussion

### 3.1. Application of PGPF Improved Herb Yield and Quality

In the present study, A13 promoted the accumulation of effective components in the roots of *Salvia miltiorrhiza*, with increases in the total phenolic acid, LAA and LAB contents of 210, 128 and 213%, respectively. In particular, the promotion of the LAB content by A13 was most significant at an A13 dose of more than twice of that of the control. In contrast, in a previous study, treatment with a yeast elicitor increased LAB production by only 115% [4]. More importantly, the effects of A13 on growth and the accumulation of medicinal ingredients was notable and stable in both the greenhouse and field trials. Thus, the prospects of A13 as a bio-fertiliser applied during *S. miltiorrhiza* planting are substantial and wide-ranging. Compared with abiotic elicitors, biotic elicitors have significant advantages for the accumulation of bioactive compounds [24]. Among biotic elicitors, co-culturing with the live bacterium *Bacillus cereus* induced a greater production of effective ingredients by *S. miltiorrhiza* hairy roots compared with roots treated with bacterial water extract or bacterial culture supernatant [9]. The application of endophytes in medicinal plant cultivation has been neglected and undervalued, but studies on the effects of co-cultivation of medicinal plants with live endophytes are progressing gradually. For example, *Piriformospora indica* enhanced asiaticoside production in tissue culture seedlings of *Centella asiatica* [14], *Mycena* sp. F-23 stimulated the kinsenoside and flavonoid contents of *Anoectochilus formosanus* in pot cultures [15], and *Mycena* sp. MF23 promoted dendrobine biosynthesis by *Dendrobium nobile* in tissue culture seedlings [25]. In an 18-month field experiment of *Dendrobium officinale*, *Mycena* sp. M2 inhibited plant growth in the early stage, but significantly increased the survival rate and dry weight after establishing a symbiosis [26]. In summary, endophytes play an important role in the synthesis and accumulation of active ingredients in medicinal plants.

### 3.2. The Changing Law of Enzyme Activities to Guide Agronomic Measures for Active Components Accumulation

**Figure 6:**
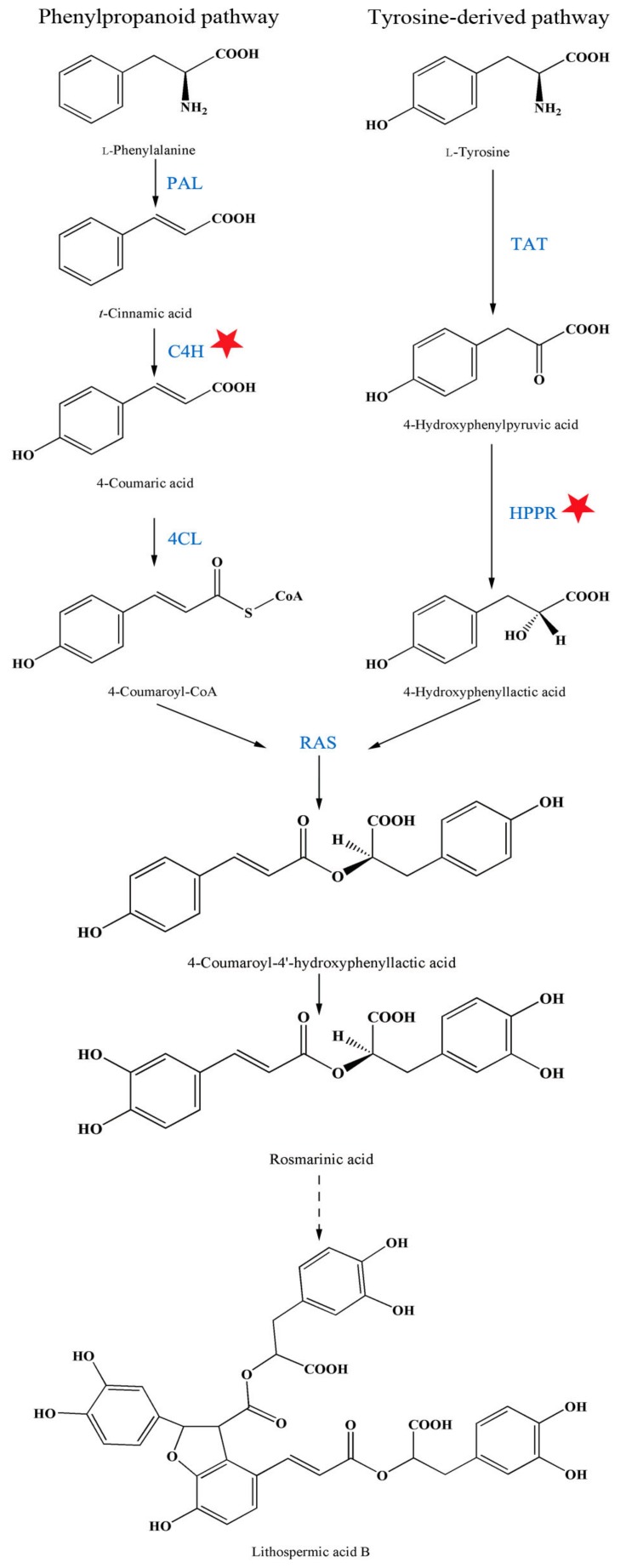
The metabolic pathways leading to LAB. PAL, phenylalanine ammonia-lyase; C4H, cinnamic acid 4-hydroxylase; TAT, tyrosine aminotransferase; HPPR, 4-hydroxyphenylpyruvate reductase. Red star indicate the enzyme which been affected significantly by A13.

By depicting gene-to-metabolite networks based on canonical correlation analysis of transcripts and specific metabolites from elicited *S. miltiorrhiza* hairy root cultures, Xiao [27] found that the C4H and HPPR transcripts were most closely correlated with phenolic acid accumulation. The contents of LAB in *C4H*-transgenic lines and *HPPR*-transgenic lines were increased by 11.1-fold and 12.7-fold compared to the control, respectively [28]. Hence, C4H and HPPR play key roles in the pathway of LAB biosynthesis. Our enzyme activity determination results showed that A13 promoted LAB accumulation, with significant enhancement of C4H and HPPR activities (Figure 6). However, only the change in C4H activity corresponded with the LAB content. Cinnamic acid 4-hydroxylase catalyses the second reaction of the phenylpropanoid pathway, whereas HPPR catalyses the second reaction of the tyrosine-derived pathway. Thus, a significant enhancement of C4H and HPPR activities would certainly increase LAB synthesis by increasing the precursor production in the two parallel pathways. Corresponding to the decrease in C4H activity at 6 months after A13 inoculation, the content of LAB decreased due to the reduced production of 4-coumaroyl-CoA, the precursor in the phenylpropanoid pathway. After co-cultivation with A13, the enhancement of both C4H and HPPR activity promoted LAB accumulation, especially C4H activity, which acts as a bottleneck in this biosynthesis pathway. Therefore, after six months of symbiotic cultivation, suitable agronomic measures for *S. miltiorrhiza* cultivation will further improve the accumulation of active components. By analysing enzyme activities during the entire growth cycle of *S. miltiorrhiza*, changes in the active ingredients were monitored and a reasonable harvest time could be predicted.

## 4. Materials and Methods

### 4.1. Experimental Design

We collected *S. miltiorrhiza* samples inhabiting wild, semi-wild, farmland and pot-cultured areas (Supplementary Figure S1) in the Henan province of China (33°38′ N, 113° E). The endophytes were isolated from roots, shoots and leaves, using the fresh tissue separation method. The fresh tissues were surface-sterilized in 70% ethanol for 1 min, followed by 2.5% NaClO for 15 min and rinsing with sterile distilled water three times. The tissues were then sectioned using a sterile blade and were placed and cultured on potato dextrose agar (PDA).After transferring the hyphal tips from each tissue section to fresh PDA several times, the endophytes were purified and obtained [29]. The strains were identified by phylogenetic analysis of the internally transcribed spacer (ITS) regions of their ribosomal DNA (rDNA) by MEGA 7.01, using the primers ITS1(5′-TCC GTAGGTGAACCTGCGG3′) and ITS4(5′-TCCTCCGCTTATTGATATGC-3′) [30]. Polymerase chain reaction (PCR) was used with the following program: initial denaturation at 95 °C for 3 min; 35 cycles of 1 min of denaturation at 94 °C, 30 s of primer annealing at 55 °C, extension at 72 °C for 1 min and a final extension at 72 °C for 7 min. After purification and sequencing of the PCR products by Genewiz Biological Engineering Technology & Services (Beijing, China), the sequence of each strain was obtained. Using the NCBI BLASTn program, sequences similar to the ITS regions of each strain were retrieved. When the similarity level between the ITS sequence of the strain and that of the sequences obtained via BLASTn searches was greater than 98%, the strain was assigned tothe same genus as that of the closest sequence. Twenty-eight candidates (Supplementary Table S1) of the dominant genus *Alternaria* in *S. miltiorrhiza* were selected for screening as PGPF for *S. miltiorrhiza* cultivation.

Greenhouse experiments were performed to initially screen the 28 candidates and to evaluate their effects on the total phenolic acid accumulation of *S. miltiorrhiza* roots collected after 6 months of cultivation. Subsequently, the effects of the screened active strains on the dry weight and LAA and LAB contents of *S. miltiorrhiza* roots were further tested over a period of six months under greenhouse conditions. The strain with optimal results in the first two tests was further examined for its effect on growth, active composition accumulation and active ingredient biosynthetic enzyme activities in *S. miltiorrhiza* roots under field conditions. Samples were collected at 2, 4 and 6 months. The interaction of the strains and *S. miltiorrhiza* roots was observed via a scanning electron microscope (SEM) as follows. After one month of co-culturing, the control and A13-colonised root tissues were fixed with 2.5% glutaraldehyde in phosphate buffer (pH 7.2) and dehydrated by passage for 30 min each in 30%, 50%, 70%, 80%, 95%, and finally 100% ethanol twice, followed by a 30-min incubation in tertiary butyl alcohol thrice at 38 °C. Freeze-dried samples were mounted on a specimen stub using double-sided adhesive tape, coated with platinum and analysed using a JSM-6510LV low vacuum scanning electron microscope (JEOL, Kyoto, Japan). 

### 4.2. Fungal Materials

To prepare the primary inoculum, each fungus was cultured on potato dextrose agar (PDA) medium in the dark at 25 °C for 10 days. Subsequently, fungal hyphae from the primary inocula were transferred to solid medium containing wheat bran and wood shavings (2:3, *v*/*v*) and were continuously grown in the dark at 25 °C for 4 weeks to generate solid inocula.

### 4.3. Greenhouse and Field Experiments

River sand and dried brown loam soil (Institute of Medicinal Plant Development campus field soil), mixed in a 1:2 ratio, were prepared to screen the growing plants under greenhouse conditions. Thirty-day-old rooting plantlets of *S. miltiorrhiza* were inoculated with 1 g of candidate fungal isolate solid inoculum, which was placed near the plant caudexes on the medium. Controls were established using equal-sized solid medium without fungus. All treatments were performed in triplicate (Supplementary Figure S2A). Root samples were collected after 6 months, separated, washed with distilled water and oven dried for 1 week at 50 °C.

A well-drained field (experimental plot at the Institute of Medicinal Plant Development experimental plot, Beijing) containing brown loam soil was left unused for several years. Thirty-day-old rooting plantlets of *S. miltiorrhiza* were inoculated with 1 g each of an A13 solid inoculum, which was placed near the plant caudexes on the medium. Controls were established using equal-sized solid medium without fungus. After 1 month of pot culturing, the inoculated plantlets were transferred to the test field with a 30 × 30 cm plant density (Supplementary Figure S2B). All treatments were performed in duplicate. Growth promotion and yield parameters were determined after 2, 4 and 6 months of co-culture. Roots were separated and washed with distilled water. For determination of the root dry weight of the plantlets, the materials were oven-dried for 1 week at 50 °C.

### 4.4. Extraction and Analysis of the Active Ingredients

Two hundred milligrams of dried root materials were extracted with 50 mL of water for 2 h by shaking at 120× rpm at 35 °C. After centrifugation (12,000× rpm, 2 min, 4 °C), 200 μL of supernatant were collected in a 1.5-mL plastic centrifuge tube. Then, the supernatant was mixed with 250 μL of 1% sodium nitrite and was incubated for 6 min. The mixture was combined with 25 μL of 20% aluminium nitrate and was incubated for another 6 min. Subsequently, the reaction was mixed with 400 μL of 1 M NaOH and fixed in 1 mL of water. After standing for 15 min, the reaction mixture was read at 506 nm with an Enspire multimode reader (PerkinElmer, Waltham, MA, USA). The total phenolic acid content was then calculated according to a standard curve, using LAB as a reference substance, the concentration of which was the highest among all the other phenolic acids in *S. miltiorrhiza* roots. By UV spectrum scanning from 250 to 550 nm, the maximum absorption peak of the reference substance was at 506 nm.

Subsequently, 200 mg of dried root material were extracted with 50 mL of water for 2 h by shaking at 120× rpm at 35 °C. After centrifugation (12,000× rpm, 2 min, 4 °C), the supernatant was collected and filtered using a 0.45-μm diameter microporous membrane for analysis. Concentrations of LAA and LAB were quantified via high-performance liquid chromatography (HPLC) using a Waters Phenomenex ODS column (250 × 4.6 mm, 4 μm) with methanol: acetonitrile: methanoic acid: water (30:10:0.5:59.5) as the mobile phase. The injection volume was 20 μL, with a column temperature of 25 °C. The detection wavelength was set at 286 nm, and an isocratic elution was used at a flow rate of 1 mL min^−1^. Qualification and quantification analyses were based on comparisons with LAA and LAB standards. The LAA and LAB peaks in the fresh samples were identified by comparing their retention times and areas with those of the matching standard. 

### 4.5. Enzyme Activity Analysis

Control and A13-colonised root tissues were sampled at 2, 4, and 6 months, using a pair of sterilised scissors, and immediately frozen in liquid nitrogen and stored at −80 °C until use. The PAL activity was measured as described by Mozzetti [31]. The TAT activity was determined using a method slightly modified from those of Yan [19] and Diamondstone [32]. The C4H activity was determined using a slight modification of the methods described by Lamb [33] and Koopmann [34]. The HPPR activity was determined via a slightly modified method of Mizukami [35].

### 4.6. Statistical Analysis

Each experiment was performed in triplicate, and the entire experimental setup was repeated in triplicate with other batches of plant material to examine the reproducibility. The means and standard deviations (SDs) were calculated using the software package SPSS Statistics 17.0 software (SPSS Inc., Chicago, IL, USA). An independent samples *t* test was used for statistical evaluations between each treatment and the control.

## 5. Conclusions

We isolated an endophytic fungus, A13, that can significantly promote the growth and medicinal ingredient accumulation of *S. miltiorrhiza* under greenhouse and field conditions. Moreover, the PGPF A13 affects the LAB content by influencing the C4H activity in the phenylpropanoid pathway, one of two parallel pathways related to LAB biosynthesis. The fungus A13 has been patented in China (Patent Number: ZL2013.1.0050774.0), and is preserved at the China General Microbiological Culture Collection Center (CGMCC; No. 6380). Therefore, A13 can be used as a potent bio-fertiliser for *S. miltiorrhiza* cultivation to improve plant quality and yield. The excellent performance of A13 supports its broad development prospects as well as great value as a solution to the lack of raw herbal materials. The fungus A13 will enhance the scope and acreage of *S. miltiorrhiza* cultivation, especially in non-origin areas, while maintaining good plant quality. To achieve this aim, we will further expand our test plots to include the cultivation of *S. miltiorrhiza* in origin and non-origin regions in the next stage of research.

## Supplementary Materials

Supplementary materials can be found at www.mdpi.com/1422-0067/19/1/270/s1.
