# Supplementary material for: The Plant Growth-Promoting Fungus (PGPF) Alternaria sp. A13 Markedly Enhances Salvia miltiorrhiza Root Growth and Active Ingredient Accumulation under Greenhouse and Field Conditions
Source: Int J Mol Sci. 2018 Jan 16;19(1):270. doi: 10.3390/ijms19010270 (PMC5796216; doi:10.3390/ijms19010270)
Supplement: Supplementary file 1 [file ijms-19-00270-s001.pdf]

International Journal of Molecular Sciences

Plant growth-promoting fungus (PGPF) *Alternaria* sp. A13 markedly enhances *Salvia miltiorrhiza* root growth and active ingredient accumulation under greenhouse and field conditions

Li-si Zhou[1], Kun Tang[1], Shun-xing Guo[1*]

[1]*Institute of Medicinal Plant Development, Chinese Academy of Medical Sciences &Peking Union Medical College, Beijing 100193, China*

*E-mail:sxguo1986@163.com TEL&FAX:86-010-57833231

Supplementary Information

Table S1 Essential information of the 28 candidates.

Fig. S1 Inhabitats of *Salvia miltiorrhiza.*

Fig. S2 Greenhouse and field experiment of *Salviae miltiorrhiza*.

Table S1 Essential information of the 28 candidates

Isolates	Habitat	Tissue	GenBank Accession No.
Alternaria sp. A1	semi-wild	root	MG515036
Alternaria sp. A2	semi-wild	root	MG515047
Alternaria sp. A3	semi-wild	root	MG515057
Alternaria sp. A4	pot-culture	root	MG515058
Alternaria sp. A5	pot-culture	root	MG515059
Alternaria sp. A6	pot-culture	root	MG515060
Alternaria sp. A7	pot-culture	root	MG515061
Alternaria sp. A8	pot-culture	root	MG515062
Alternaria sp. A9	pot-culture	root	MG515063
Alternaria sp. A10	pot-culture	root	MG515037
Alternaria sp. A11	farmland	root	MG515038
Alternaria sp. A12	farmland	root	MG515039
Alternaria sp. A13	farmland	root	MG515040
Alternaria sp. A14	wild (>2 years)	shoot	MG515041
Alternaria sp. A15	wild (>2 years)	shoot	MG515042
Alternaria sp. A16	pot-culture	shoot	MG515043
Alternaria sp. A17	pot-culture	shoot	MG515044
Alternaria sp. A18	wild	leaf	MG515045
Alternaria sp. A19	wild (>2 years)	leaf	MG515046
Alternaria sp. A20	wild (>2 years)	leaf	MG515048
Alternaria sp. A21	wild (>2 years)	leaf	MG515049
Alternaria sp. A22	semi-wild	leaf	MG515050
Alternaria sp. A23	pot-culture	leaf	MG515051
Alternaria sp. A24	pot-culture	leaf	MG515052
Alternaria sp. A25	pot-culture	leaf	MG515053
Alternaria sp. A26	pot-culture	leaf	MG515054
Alternaria sp. A27	farmland	leaf	MG515055
Alternaria sp. A28	farmland	leaf	MG515056

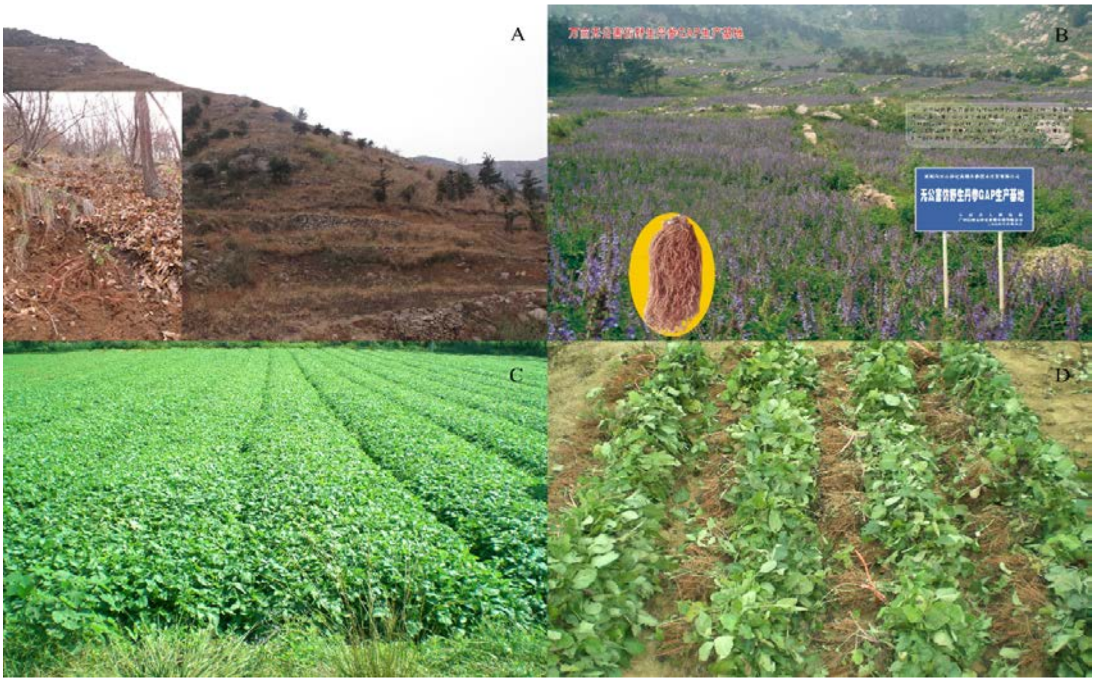

Fig. S1 Inhabitats of *Salvia miltiorrhiza.*

Salvia miltiorrhiza inhabited wild(A), semi-wild(B), farmland(C) and pot-cultured(D).

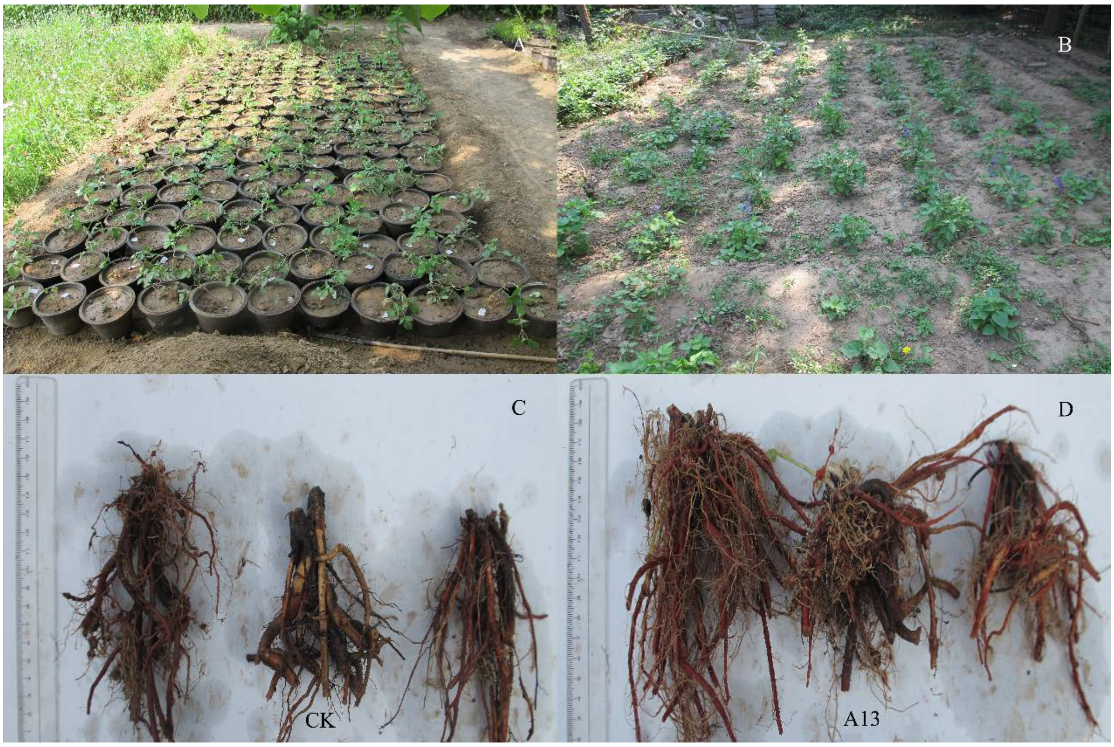

Fig. S2 Greenhouse and field experiment of *Salviae miltiorrhiza*.

Greenhouse experiment after inoculation 0-month(A); Field experiment after inoculation 4-months(B); *Salviae miltiorrhiza* roots without inoculation cultured for 6-months in the field(C); *Salviae miltiorrhiza* roots after inoculation with A13 cultured for 6-months in the field(D) .
